# Reference genes for the developing mouse lung under consideration of biological, technical and experimental confounders

**DOI:** 10.1038/s41598-022-19071-1

**Published:** 2022-10-21

**Authors:** H. Shin, R. E. Morty, J. M. Sucre, N. M. Negretti, M. Markmann, H. Hossain, S. Krauss-Etschmann, S. Dehmel, A. Hilgendorff

**Affiliations:** 1 Institute for Lung Biology and Disease and Comprehensive Pneumology Center, Helmholtz Zentrum München, Member of German Center for Lung Research (DZL), Munich, Germany; 2 Department of Translational Pulmonology, University Hospital Heidelberg, Heidelberg, Germany; 3 Translational Lung Research Center, member of the German Center for Lung Research (DZL), Heidelberg, Germany; 4 Department of Pediatrics, Vanderbilt University Medical Center, Nashville, TN USA; 5 Department of Anesthesiology, Intensive Care Medicine and Pain Therapy, Justus-Liebig-University, Giessen, Germany; 6 Institute of Laboratory Medicine and Microbiology, Klinikum St. Marien Amberg and Kliniken Nordoberpfalz AG, Weiden, Germany; 7 Center for Comprehensive Developmental Care (CDeCLMU), University Hospital, Ludwig-Maximilians-University, Munich, Germany; 8 Present Address: Priority Area Chronic Lung Diseases, Early Life Origins of Chronic Lung Disease, Research Center Borstel, Leibniz Lung Center, German Center for Lung Research (DZL) and the Airway Research Center North (ARCN), Borstel, Germany; 9 Present Address: Institute for Experimental Medicine, Christian Albrechts University, German Center for Lung Research (DZL) and the Airway Research Center North (ARCN), Kiel, Germany; 10 Present Address: Strategy, Programs, Resources (SPR), Helmholtz Zentrum München, Munich, Germany

**Keywords:** Reverse transcription polymerase chain reaction, Respiratory tract diseases

## Abstract

For gene expression analysis, the raw data obtained from RT-qPCR are preferably normalized to reference genes, which should be constantly expressed regardless of experimental conditions. Selection of reference genes is particularly challenging for the developing lung because of the complex transcriptional and epigenetic regulation of genes during organ maturation and injury repair. To date, there are only limited experimental data addressing reliable reference genes for this biological circumstance. In this study, we evaluated reference genes for the lung in neonatal C57BL/6 mice under consideration of biological, technical and experimental conditions. For that, we thoroughly selected candidates from commonly used reference genes side-by-side with novel ones by analyzing publicly available microarray datasets. We performed RT-qPCR of the selected candidate genes and analyzed their expression variability using GeNorm and Normfinder. Cell-specific expression of the candidate genes was analyzed using our own single-cell RNA-sequencing data from the developing mouse lung. Depending on the investigated conditions, i.e., developmental stages, sex, RNA quality, experimental condition (hyperoxia) and cell types, distinct candidate genes demonstrated stable expression confirming their eligibility as reliable reference genes. Our results provide valuable information for the selection of proper reference genes in studies investigating the neonatal mouse lung.

## Introduction

Real-time quantitative PCR (RT-qPCR) is a commonly used method for the quantification of gene expression levels due to its numerous advantages, including high sensitivity and specificity, reliable reproducibility and significant accuracy even in low amount samples^1^. With the increasing application of RT-qPCR in different research areas, the role of so-called reference genes becomes more important to allow for the accurate interpretation of experimental data and the comparability across replicates. Relative quantification, i.e., normalization of the expression level of a gene of interest to a reference gene used as an endogenous controls compensates gene expression variability induced by experimental conditions such as differences in sample volume, RNA integrity or cDNA synthesis.

In order to ensure robust performance, reference genes need to meet critical quality criteria including (i) consistent expression levels in the investigated tissue, (ii) resilience toward tissue processing conditions and (iii) robustness to experimental challenges imposed on the tissues investigated^2,3^. These prerequisites are especially challenged in the developing organ undergoing rapid and significant functional and structural changes. After birth, the mouse lung undergoes alveolarization while being exposed to the environment and its different conditions. This not only renders the lung as a prime target to study repair and regeneration processes under clinically relevant conditions, but marks a challenging condition for the selection of reference genes at the same time due to the ever-changing portfolio of pathways and cellular crosstalk during postnatal development.

Despite this need, little is known about reliable reference gene candidates. Previous studies demonstrated that commonly used reference genes are significantly affected by tissue origin, developmental stages and experimental conditions^4^.

To address this challenge, we have used several complimentary tools to perform a comprehensive evaluation of pre-selected reference gene candidates in neonatal lung tissue targeting their expression variability across critical biological, technical and experimental conditions.

## Results

### Identification of potential reference gene candidates by public and commercial databases

Reference gene candidates were pre-selected using the four data sources a–d as described in the methods and summarized in one comprehensive candidate list (n = 122 genes before considering overlaps, Supplementary Table S1):

- PubMed search revealed seven reference genes from six RT-qPCR studies performed in lung tissue of C57BL/6 mice^5–10^.
- Four studies including one meta-analysis evaluating reference genes in murine lung tissue and other organs^11^ identified 30 reference gene candidates^11–14^.
- Genevestigator analysis revealed 58 reference gene candidates considering three different expression levels (range of gene expression variability: SD 0.16–0.96).
- The commercial reference gene catalogue (Roche RealTime Ready panel) included 27 reference genes.

In a subsequent step, 27 genes were excluded according to one or more of the following criteria: overlap between selection steps (n = 14) or presence of pseudogenes (n = 12; www.informatics.jax.org). Due to conflicting findings regarding the eligibility of ribosomal genes as reference genes^11,15^, n = 10 genes coding for ribosomal proteins were excluded leaving one representative candidate for evaluation (*Rpl4*). *Hbb-b1,* coding for a subunit of the oxygen carrier was intentionally excluded as regulation under hyperoxia conditions has to be expected. This resulted in a final list of n = 95 reference gene candidates.

### Selection of final reference gene candidates and validation of expression variability by RT-qPCR

Pulmonary expression of 46 out of 95 reference gene candidates was confirmed in our own transcriptome data from neonatal mice. Of these, 20 reference gene candidates yielding the lowest overall SD (0.118–0.242) in our own transcriptome data and in the Genevestigator dataset were selected for further analysis. As the majority of these candidates (18 of 20 genes) derives from Genevestigator, four additional candidates were randomly selected from the candidate genes ranking 21–46 according to the overall SD in order to broaden the spectrum of data sources considered (Table 1).

**Table 1:** The 24 final reference gene candidates.

Gene symbol	Entrez ID	Source	Expression level	SD*ref*	SD*array*	SD*overall*	Rank
***Dolpp1***	57170	c	Medium	0.16	0.05	0.118	1
***Mpv17l***	93734	c	Medium	0.18	0.03	0.129	2
***Elac2***	68626	c	Medium	0.19	0.05	0.139	3
***Pcsk7***	18554	c	Medium	0.20	0.03	0.143	4
***Kdm3b***	277250	c	Medium	0.19	0.07	0.143	5
***Maea***	59003	c	Medium	0.21	0.05	0.152	6
Fkbp1a*	14225	c	High	0.24	0.06	0.174	7
***Wars2***	70560	c	Medium	0.24	0.08	0.179	8
***Auh***	11992	c	Low	0.24	0.08	0.180	9
***Efhd2***	27984	c	High	0.27	0.02	0.192	10
***Zc3h11a***	70579	c	High	0.28	0.02	0.198	11
***Rexo5***	434234	c	Low	0.28	0.03	0.199	12
***Nupl2***	231042	c	Low	0.27	0.10	0.204	13
***Tmed2***	56334	c	High	0.31	0.02	0.220	14
***Grn***	14824	c	High	0.33	0.02	0.234	15
***Eif3a***	13669	b	Low	0.32	0.09	0.235	16
***Tbp***	21374	d	Medium	0.33	0.04	0.235	17
***Csnk1a1***	93687	c	High	0.33	0.08	0.241	18
***Eif4g2***	13690	c	High	0.34	0.03	0.241	19
***Tnks2***	74493	c	High	0.34	0.04	0.242	20
*F2r*	14062	c	High	0.32	0.13	0.243	21
*Rab23*	19335	c	Medium	0.34	0.10	0.251	22
*Myadm*	50918	c	High	0.33	0.15	0.256	23
*Rrp1b*	72462	c	Medium	0.37	0.07	0.266	24
*Hprt*	15452	d	High	0.37	0.07	0.267	25
*Canx*	12330	c	High	0.35	0.15	0.268	26
*Eif4h*	22384	c	High	0.39	0.04	0.277	27
*Slc35a5*	74102	c	High	0.41	0.03	0.291	28
*Atp6v0c*	11984	c	High	0.45	0.06	0.321	29
*Ywhaz*	22631	d	High	0.45	0.06	0.321	30
*Mx2*	17858	d	Low	0.43	0.17	0.328	31
*Hdgfrp2*	15193	c	Medium	0.52	0.00	0.368	32
***Puf60***	67959	b	High	0.54	0.01	0.382	33
***Tfrc***	22042	d	High	0.51	0.21	0.390	34
*Tomm20*	67952	c	High	0.56	0.04	0.397	35
***Psmd4***	19185	b	High	0.62	0.00	0.438	36
*Sdha*	66945	d	High	0.69	0.06	0.490	37
*G6pdx*	14381	d	High	0.71	0.04	0.503	38
*Rhot1*	59040	c	High	0.53	0.48	0.504	39
*Ing3*	71777	c	Medium	0.78	0.05	0.553	40
*Gusb*	110006	a, b, d	Medium	0.79	0.01	0.559	41
*Pla2g2a*	18780	d	Low	0.80	0.03	0.566	42
*Actb*	11461	a, b, d	High	0.83	0.04	0.588	43
***Rpl4***	67891	b	High	0.85	0.02	0.601	44
*Gapdh*	14433	a, b, d	High	0.88	0.05	0.623	45
*Hsp90ab1*	15516	d	High	0.92	0.47	0.730	46

After the exclusion of 27 genes according to the criteria outlined in the text and subsequent confirmation of gene expression in our own transcriptomic data, 46 out of 122 reference gene candidates, shown in the table, remained for further analysis. Next, these 46 genes were ranked according to the overall SD covering both microarray data from our own and Genevestigator. The top 20 candidates with lowest overall SD and four additional genes chosen randomly from the rank 21–46 were selected for validation in neonatal murine lungs by RT-qPCR. Finally, it resulted in the 24 final reference gene candidates (highlighted in bold letters): four genes from reference gene studies, 18 genes from Genevestigator and two genes from the Roche commercial panel. *Fkbp1a was later excluded from further raw data analysis due to its undetectable low expression levels in several RNA samples. Gene expression levels are represented as log2-scaled signal intensity according to Genevestigator analysis: > 13.00 for high level, 10.00–13.00 for medium level, < 10.00 for low level. SDref stands for the SD from RefGenes analysis of Genevestigator and SDarray for the SD from our own microarray data. The overall SD was calculated by averaging SDref and SDarray. The source a for commonly used reference gene, b for reference gene study, c for Genevestigator and d for Roche reference gene panel.

In the next step, expression variability of all 24 reference gene candidates was validated by RT-qPCR. Expression of *Fkbp1a* was not detectable in 6 of 60 samples (no measurable Cq values) and was therefore excluded from further analysis to guarantee reliable expression of the selected reference genes. This finding was potentially related to the low RNA quality (RIN < 5) in these samples. It resulted in a total of 23 final reference gene candidates (exclusion of *Fkbp1a* from the 24 initially selected genes). Furthermore, *Psmd4* showed low expression levels in 13 out of 86 samples, so that the suitability of this gene as a reference gene likely depends on the particular experimental setting, which needs to be considered with caution.

### Expression variability of the reference gene candidates under physiologic condition with relation to developmental stage

In order to address expression variability in the neonatal mouse lung, we first characterized expression variability using all lung samples obtained under physiologic conditions (n = 17, PND2.5–28.5, FiO_2_ = 0.21, RIN > 5). Here, GeNorm identified 7 out of the 23 reference gene candidates with acceptable gene stability values (M-value < 0.5)^16^. Among them, *Nupl2* and *Rpl4* showed the lowest expression variability (M-value 0.280 for both) followed by *Csnk1a1*, *Maea*, *Eif3a*, *Elac2* and *Dolpp1* with increasing M-values. Likewise, Normfinder identified 8 out of the 23 reference gene candidates with acceptable gene stability values (SV < 0.5)^17^ and among them, *Maea* demonstrated the lowest expression variability (SV = 0.205) followed by *Csnk1a1*, *Nupl2*, *Rpl4*, *Zc3h11a*, *Eif3a*, *Pcsk7* and *Dolpp1* with increasing SV values (Tables 2, 3).

**Table 2:** GeNorm result represented with M-values and corresponding stability ranking for different analytic approaches compared with the pre-selection.

Reference gene candidates	Pre-selection		Physiologic condition (FiO_2_ = 0.21, PND2.5–28.5, RIN > 5; n = 17)		Developmental stages (FiO_2_ = 0.21, RIN > 5)						Sex (FiO_2_ = 0.21, PND2.5–28.5, RIN > 5)				Low RNA quality (FiO_2_ = 0.21, PND2.5–28.5, RIN < 5; n = 33)		Hyperoxia exposure (FiO_2_ = 0.8, PND2.5–28.5, RIN > 5; n = 36)	
					PND2.5 (n = 6)		PND5.5 (n = 7)		PND14.5–28.5 (n = 4)		Male (n = 31)		Female (n = 29)					
	Overall SD	Rank	M-value	Rank	M-value	Rank	M-value	Rank	M-value	Rank	M-value	Rank	M-value	Rank	M-value	Rank	M-value	Rank
*Auh*	0.151	8	0.541	10	0.204	8	0.463	13	0.480	16	0.392	8	0.397	7	1.537	22	0.299	1
*Csnk1a1*	0.199	19	0.375	3	0.317	17	0.382	9	0.420	11	0.231	1	0.224	1	0.908	11	0.407	5
*Dolpp1*	0.113	1	0.498	7	0.125	4	0.132	1	0.558	21	0.416	10	0.418	8	0.640	5	0.372	4
*Efhd2*	0.157	9	0.589	13	0.265	13	0.295	5	0.532	20	0.433	12	0.465	11	0.585	4	0.601	13
*Eif3a*	0.197	16	0.431	5	0.355	20	0.429	12	0.490	17	0.333	5	0.510	15	0.541	3	0.535	10
*Eif4g2*	0.198	17	0.836	20	0.236	11	0.619	17	0.347	8	0.231	1	0.312	4	0.469	1	0.583	12
*Elac2*	0.119	4	0.468	6	0.061	1	0.284	4	0.303	7	0.425	11	0.438	9	1.099	15	0.496	8
*Grn*	0.191	14	0.937	22	0.097	3	0.814	21	0.376	9	0.446	14	0.478	12	1.259	18	0.890	22
*Kdm3b*	0.121	5	0.555	11	0.165	6	0.253	3	0.500	18	0.476	17	0.520	16	0.469	1	0.745	18
*Maea*	0.137	6	0.401	4	0.292	15	0.412	11	0.239	5	0.369	7	0.490	13	0.669	6	0.436	6
*Mpv17I*	0.113	2	1.053	23	0.509	23	0.966	23	0.737	23	0.719	23	0.782	23	1.619	23	0.950	23
*Nupl2*	0.178	12	0.280	1	0.141	5	0.314	6	0.221	4	0.451	15	0.453	10	0.766	8	0.510	9
*Pcsk7*	0.118	3	0.513	8	0.061	1	0.132	1	0.514	19	0.406	9	0.542	18	0.868	10	0.474	7
*Psmd4*	0.358	22	0.568	12	0.278	14	0.334	7	0.189	3	0.440	13	0.575	20	0.704	7	0.562	11
*Puf60*	0.312	20	0.884	21	0.414	22	0.881	22	0.586	22	0.615	22	0.667	22	1.453	21	0.843	21
*Rexo5*	0.166	11	0.714	16	0.343	19	0.505	14	0.401	10	0.514	19	0.597	21	0.971	12	0.773	19
*Rpl4*	0.491	23	0.280	1	0.222	10	0.360	8	0.152	1	0.535	20	0.531	17	1.320	19	0.340	3
*Tbp*	0.194	15	0.637	14	0.215	9	0.551	15	0.469	15	0.265	3	0.269	3	1.138	16	0.669	15
*Tfrc*	0.333	21	0.778	18	0.304	16	0.771	20	0.261	6	0.354	6	0.337	5	1.016	13	0.722	17
*Tmed2*	0.185	13	0.806	19	0.248	12	0.723	19	0.152	1	0.566	21	0.224	1	1.196	17	0.797	20
*Tnks2*	0.198	18	0.748	17	0.372	21	0.665	18	0.444	13	0.462	16	0.555	19	1.059	14	0.700	16
*Wars2*	0.150	7	0.676	15	0.189	7	0.585	16	0.459	14	0.492	18	0.500	14	1.384	20	0.631	14
*Zc3h11a*	0.166	10	0.528	9	0.331	18	0.400	10	0.432	12	0.313	4	0.368	6	0.827	9	0.299	1

**Table 3:** Normfinder result represented with SV-values and corresponding stability ranking for different analytic approaches compared with the pre-selection.

Reference gene candidates	Pre-selection		Physiologic condition (FiO_2_ = 0.21, PND2.5–28.5, RIN > 5; n = 17)		Developmental stages (FiO_2_ = 0.21, RIN > 5)						Sex (FiO2 = 0.21, PND2.5–28.5, RIN > 5)				Low RNA quality (FiO_2_ = 0.21, PND2.5–28.5, RIN < 5; n = 33)		Hyperoxia exposure (FiO_2_ = 0.8, PND2.5–28.5, RIN > 5; n = 36)	
					PND2.5 (n = 6)		PND5.5 (n = 7)		PND14.5–28.5 (n = 4)		Male (n = 31)		Female (n = 29)					
	Overall SD	Rank	SV	Rank	SV	Rank	SV	Rank	SV	Rank	SV	Rank	SV	Rank	SV	Rank	SV	Rank
*Auh*	0.151	8	0.553	10	0.201	7	0.755	16	0.541	17	0.383	9	0.229	1	2.262	22	0.301	3
*Csnk1a1*	0.199	19	0.278	2	0.217	9	0.275	3	0.314	8	0.286	5	0.393	9	0.817	6	0.333	6
*Dolpp1*	0.113	1	0.496	8	0.403	18	0.366	4	0.808	22	0.342	7	0.402	10	0.611	4	0.322	5
*Efhd2*	0.157	9	0.810	15	0.506	20	0.717	15	0.665	19	0.508	14	0.439	12	0.970	8	0.815	17
*Eif3a*	0.197	16	0.409	6	0.271	12	0.505	9	0.418	12	0.397	10	0.273	4	0.752	5	0.476	8
*Eif4g2*	0.198	17	0.993	19	0.053	1	1.051	18	0.208	3	0.241	2	0.328	6	1.176	16	0.718	13
*Elac2*	0.119	4	0.527	9	0.376	16	0.596	14	0.125	2	0.455	13	0.486	15	1.157	14	0.523	9
*Grn*	0.191	14	1.452	22	0.336	13	1.363	21	0.270	6	0.593	19	0.570	19	1.824	20	1.390	22
*Kdm3b*	0.121	5	0.608	11	0.257	10	0.519	10	0.578	18	0.557	17	0.526	18	0.986	10	0.872	18
*Maea*	0.137	6	0.205	1	0.366	15	0.106	2	0.086	1	0.508	15	0.343	8	0.468	3	0.314	4
*Mpv17I*	0.113	2	2.444	23	1.815	23	1.902	23	2.871	23	2.266	23	2.058	23	2.320	23	1.517	23
*Nupl2*	0.178	12	0.311	3	0.216	8	0.395	6	0.254	4	0.441	12	0.415	11	0.845	7	0.465	7
*Pcsk7*	0.118	3	0.476	7	0.390	17	0.492	8	0.704	20	0.582	18	0.330	7	0.372	2	0.529	10
*Psmd4*	0.358	22	0.616	12	0.509	21	0.475	7	0.258	5	0.741	21	0.516	17	0.990	11	0.743	16
*Puf60*	0.312	20	1.341	21	0.811	22	1.537	22	0.730	21	1.372	22	1.015	22	1.920	21	1.224	21
*Rexo5*	0.166	11	0.868	17	0.259	11	0.381	5	0.336	9	0.714	20	0.582	20	1.003	12	0.884	19
*Rpl4*	0.491	23	0.329	4	0.104	5	0.522	11	0.278	7	0.364	8	0.514	16	1.449	18	0.235	1
*Tbp*	0.194	15	0.729	13	0.090	4	0.567	13	0.515	16	0.264	4	0.251	3	1.159	15	0.726	14
*Tfrc*	0.333	21	0.897	18	0.168	6	1.104	20	0.387	11	0.253	3	0.286	5	0.979	9	0.735	15
*Tmed2*	0.185	13	1.003	20	0.053	2	1.084	19	0.426	13	0.239	1	0.730	21	1.298	17	0.913	20
*Tnks2*	0.198	18	0.850	16	0.412	19	0.762	17	0.474	15	0.536	16	0.446	13	1.144	13	0.698	12
*Wars2*	0.150	7	0.774	14	0.089	3	0.545	12	0.462	14	0.406	11	0.456	14	1.675	19	0.547	11
*Zc3h11a*	0.166	10	0.368	5	0.355	14	0.106	1	0.365	10	0.302	6	0.251	2	0.297	1	0.253	2

In a second step, we characterized pulmonary gene expression variation for all 23 candidates in each developmental stage, i.e., PND2.5, 5.5 and 14.5–28.5 (FiO_2_ = 0.21; RIN > 5). Here, 9 (GeNorm) and 5 (Normfinder) genes out of the 23 reference gene candidates were identified demonstrating low variablity (M- or SV-value < 0.5) in all three groups. Among these, *Elac2* (GeNorm) and *Maea, Nupl2* (Normfinder) showed the highest mean stability ranking in all developmental stages (Tables 2, 3). Intra- and intergroup variation analysis (Normfinder) identified *Maea* (re-calculated SD 0.2207) as the reference gene with lowest variation and *Maea* together with *Nupl2* as the best combination for reference gene performance (re-calculated SD 0.1492).

### Effect of biological, experimental and technical variables on reference gene expression variability

Sex-related effects were investigated in samples spanning all developmental stages while only including RNA quality of RIN > 5 (n = 21 male, n = 20 female). Out of the 23 reference gene candidates, 16 (GeNorm) and 14 (Normfinder) genes revealed low expression variability (M- or SV-value < 0.5) in both sexes. Among these, GeNorm identified *Tbp, Tmed2* and Normfinder *Eif4g2* with the highest mean stability ranking in both sexes (Tables 2, 3). Intra- and intergroup variation analysis by Normfinder identified *Eif4g2* (re-calculated SD 0.0476) as the reference gene with the lowest variation and *Tbp* together with *Eif4g2* as the best combination for reference gene performance (re-calculated SD 0.0346).

With regard to the effect of RNA quality on expression variability of the reference gene candidates, a comparative analysis between low (n = 33; PND2.5–28.5; RIN < 5) and high (n = 17; PND2.5–28.5; RIN > 5) RNA quality samples identified no gene with an acceptable M-value of < 0.5 in both RNA quality groups (GeNorm). However, Normfinder revealed 3 out of the 23 candidate genes with low expression variability (SV < 0.5) in both RNA quality conditions. Of them, *Maea* demonstrated the highest mean stability ranking in both RNA quality conditions (Tables 2, 3).

Intra- and intergroup variation analysis by Normfinder identified *Pcsk7* as the reference gene with lowest expression variability (re-calculated SD 0.1832) and *Rpl4* and *Zc3h11a* as the best combination for reliable reference gene performance (re-calculated SD 0.1460).

In lung tissues acquired from hyperoxia-exposed mice (n = 36; PND2.5–28.5; FiO_2_ = 0.8; RIN > 5), GeNorm identified 8 out of all 23 reference gene candidates with low expression variability (M-value < 0.5) with *Auh* and *Zc3h11a* demonstrating the lowest expression variability (M-value 0.299), followed by *Rpl4*, *Dolpp1*, *Csnk1a1*, *Maea*, *Pcsk7* and *Elac2*. Likewise, Normfinder revealed 8 out of all 23 candidates with low expression variability (SV < 0.5) with *Rpl4* demonstrating the lowest expression variability (SV = 0.235) followed by *Zc3h11a*, *Auh*, *Maea*, *Dolpp1*, *Csnk1a1*, *Nupl2* and *Eif3a* (Tables 2, 3).

When comparing expression variability of all 23 candidates under physiologic (n = 17; PND2.5–28.5; FiO_2_ = 0.21, RIN > 5) and hyperoxia conditions (n = 36; PND2.5–28.5; FiO_2_ = 0.8, RIN > 5), 5 (GeNorm) and 7 (Normfinder) genes were identified with low expression variability (M- or SV-value < 0.5) in both experimental conditions. Among them, GeNorm recognized *Rpl4* and Normfinder *Maea, Rpl4* with the highest mean stability ranking in both groups, i.e., physiologic and hyperoxia conditions (Tables 2, 3).

Intra- and intergroup variation analysis by Normfinder demonstrated *Rpl4* (re-calculated SD 0.1004) as the best reference gene and *Maea* together with *Zc3h11a* as the best combination for reliable reference gene performance (re-calculated SD 0.0711).

By RT-qPCR validation, we observed differences in expression stability rankings for selected candidates, e.g., *Mpv17l*, presenting the lowest expression variability in the pre-selection in contrast to highest expression variability in RT-qPCR analysis (Tables 2, 3).

### Expression of the 23 candidates in diverse cell types in the course of neonatal development PND0-PND14

**Figure 1:**
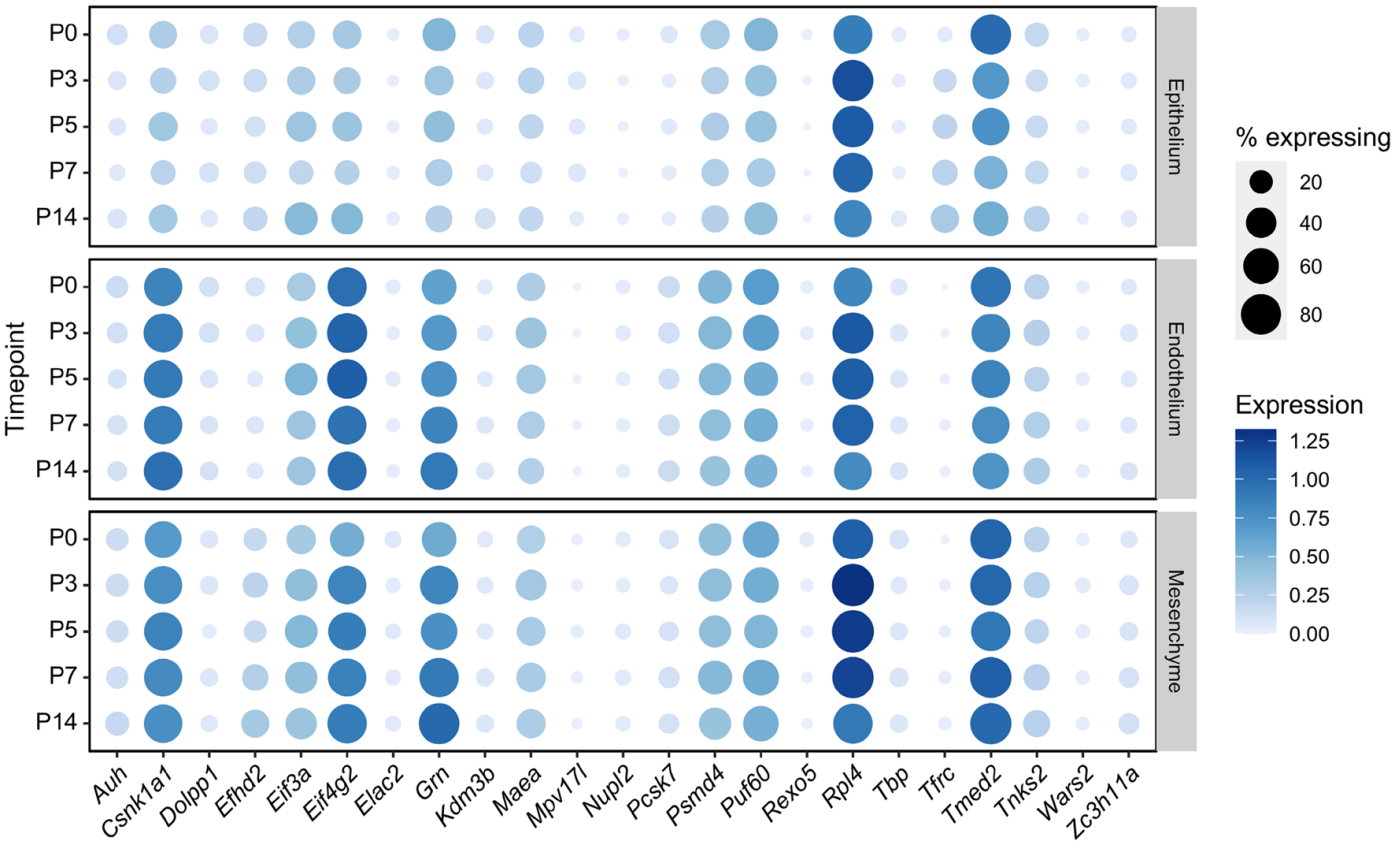
Profile of reference gene expression time in the developing lung by single-cell RNA sequencing. Using a recently published single-cell sequencing atlas of the developing mouse lung^18^, gene expression of the different candidate reference genes in broadly categorized cell types (epithelial, endothelial and mesenchymal cells) was analyzed over time. The average expression in each cell type at each timepoint is plotted in a dotplot. The size of the circle indicates the fraction of cells with expression levels above the limit of detection and the intensity of the color of the circles indicates the average expression level in each cell. Larger circles indicate a greater fraction of cells with detectable expression and darker circles indicates greater average expression.

**Figure 2:**
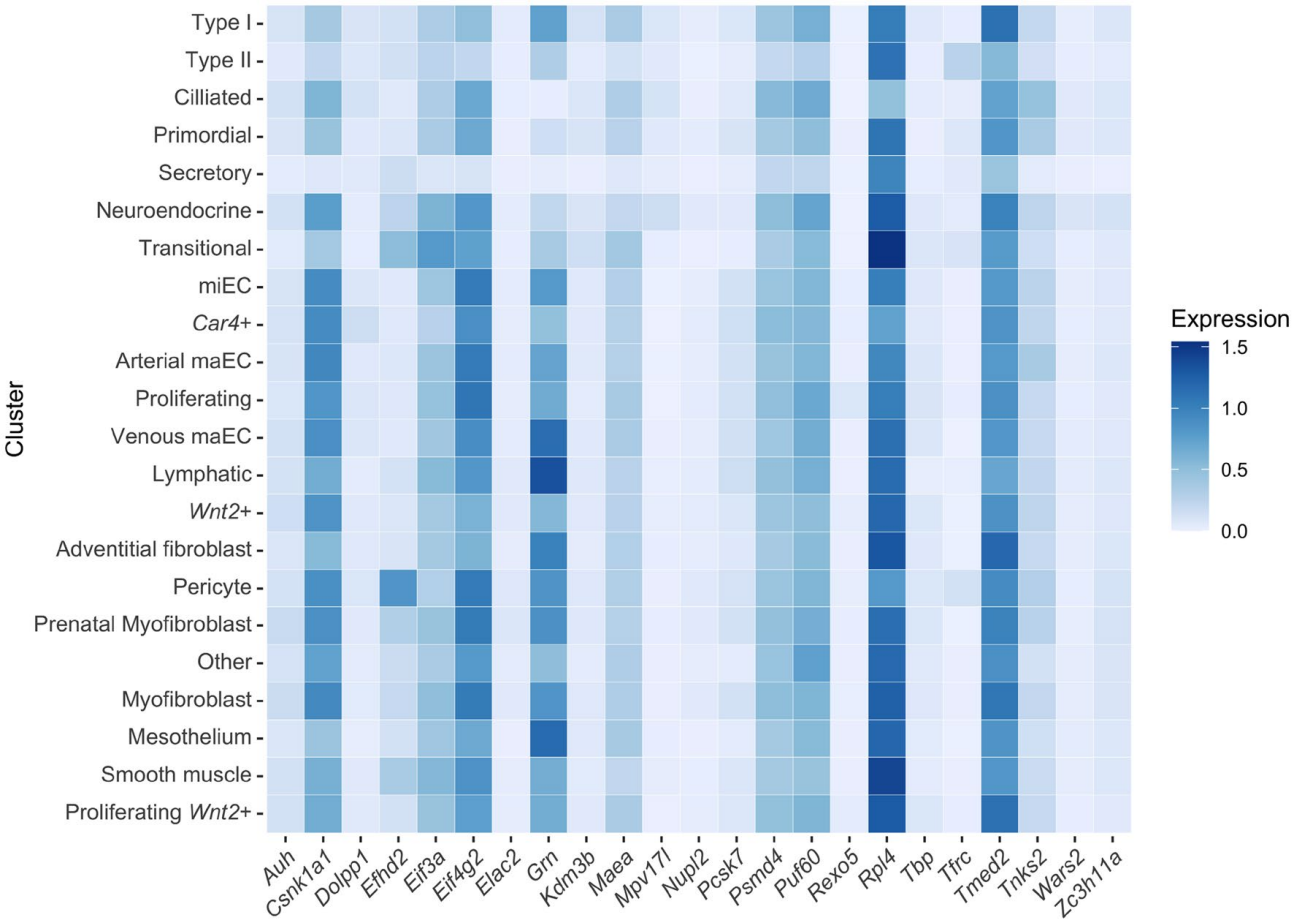
Marker gene expression was profiled in specific cell types by single-cell RNA sequencing. From the single-cell sequencing atlas of mouse lung development^18^, expression across the different cell types was averaged from mouse lungs collected at P0, P3, P5, P7 and P14 and plotted on a heatmap. Darker colors indicate greater expression.

Gene expression profiles in three major cell types (epithelial, endothelial, mesenchymal) during the course of neonatal lung development (PND0-PND14) from a recently published single-cell atlas of the developing mouse lung^18^ demonstrated *Csnk1a1*, *Eif4g2*, *Grn*, *Rpl4* and *Tmed2* as the reference gene candidates with high expression level in the majority of the investigated cells, while the former three genes showed slightly lower expression levels in the epithelium (Figs. 1, 2) The variance of each candidate gene in the scRNA-Seq dataset was calculated during SCTransform normalization and ranked from lowest to highest. The five genes with the lowest variance were: *Rexo5*, *Mpv17l*, *Wars2*, *Nupl2* and *Elac2*.

## Discussion

Accurate interpretation of experimental data obtained by RT-qPCR, i.e., presented as raw Cq values, is of critical importance for result interpretation. A widely applied method is the so-called relative quantification using reference genes as endogenous control. This normalization strategy is easy to apply and allows for compensation of non-specific gene expression variability resulting from technical and experimental conditions. These include variation due to different amount of starting material, variance in RNA integrity and inconsistent efficiency of cDNA synthesis^19–21^. For optimal performance, these reference genes need to fulfill critical criteria such as low expression variability with regard to different variables that might act as ‘hidden confounders’ when considering reference gene regulation.

We therefore thoroughly assessed important reference gene quality criteria in a clinically and experimentally relevant, challenging context presented in the developing lung. Here, organ development progresses rapidly, sample numbers are often limited and different biological, technical and experimental variables can exhibit significant effects on gene expression that need to be eliminated or taken into account when a reference gene is used. Through the application of GeNorm and Normfinder algorithms, broadly used to evaluate gene expression variability, in combination with group comparisons, we successfully characterized reference genes in the developing lungs of neonatal C57BL/6 mice. The main challenge while selecting a reference gene for studies in a developing organ, was that pre-selection of the reference gene candidates only relied on limited data from neonatal tissue. Their characterization during postnatal lung development while considering the sex and RNA quality indicated the significant impact of these conditions on reference gene expression. However, it revealed promising candidates with lower expression variability. Studies addressing the broadly used experimental condition ‘hyperoxia’ to induce lung injury further emphasized this pattern.

**Figure 3:**
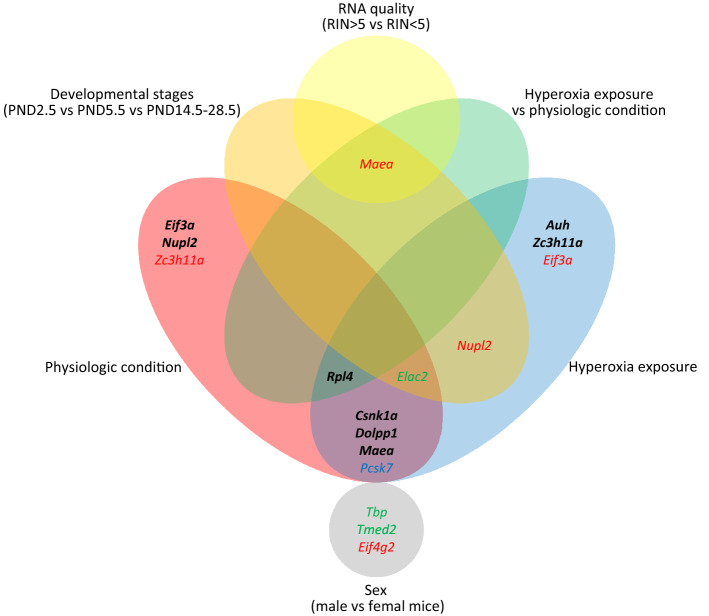
Reference genes with low expression variability (RT-qPCR). Black, bold: Genes with high expression stability identified by both GeNorm and Normfinder. Colored: Genes selected for low expression variability by GeNorm (green) oder Normfinder (red). Pcsk7 (blue) was identified by GeNorm in the hyperoxia condition and by Normfinder in the physiologic condition.

Although the analyses did not reveal one single gene with universally low expression variability in all conditions investigated, the results allowed the selection of individual genes under consideration of the different biological, technical and experimental conditions studied (summarized results Table 4, Fig. 3).

**Table 4:** Reliable reference genes identified by Normfinder intra- and intergroup variation analysis.

Investigated conditions	Normfinder intra- and intergroup variation analysis	
	Best gene	Best combination
Developmental stage	*Maea*	*Maea and Nupl2*
Sex	*Eif4g2*	*Eif4g2 and Tbp*
RNA quality	*Pcsk7*	*Rpl4 and Zc3h11a*
Hyperoxia exposure vs physiologic condition	*Rpl4*	*Maea and Zc3h11a*

The Normfinder intra- and intergroup variation analysis showed comparable results as the stability ranking analysis by Normfinder. cf. Fig. 3.

Specific considerations regarding reference gene selection reflect both, the insight generated by the study as well as the limitations of interpretability. Differences in expression stability rankings of the candidates obtained in the pre-selection process and by RT-qPCR measurements (e.g., *Mpv17l*) might reflect effects of age or analysis method and should be considered for the individual experimental design. The low expression variability, i.e., high expression stability of *Rpl4* observed in different experimental conditions has to be interpreted in light of the controversial discussion around genes encoding ribosomal proteins. The discussion is reflected by the publications of de Jonge et al.^11^, supporting reference gene qualities for this group of genes due to low expression variability, in contrast to Thorrez et al.^15^, discouraging their use as reference genes due to the signficant differences between specific tissues. In contrast to the exclusion of an entire group of genes, analysis results further required single case decisions: The lack of *Fkbp1a* expression in 10% of the neonatal lung samples resulted in the exclusion of this gene for further analysis to ensure stable reference gene expression in neonatal lung tissue. Potential effects of development on *Fkbp1a* expression were likely aggravated by low RNA quality.

Cell specific expression levels of the reference gene candidates obtained by single-cell RNA sequencing confirmed promising candidates (*Rexo5*, *Mpv17l*, *Wars2*, *Nupl2*, *Elac2*) with low expression variability in the course of lung development, i.e., PND0-PND14. These findings should be considered when addressing specific cell types or lung cellular compartments by gene expression analysis in total lung homogenates despite the overall low expression levels of these genes (Figs. 1, 2).

**Figure 4:**
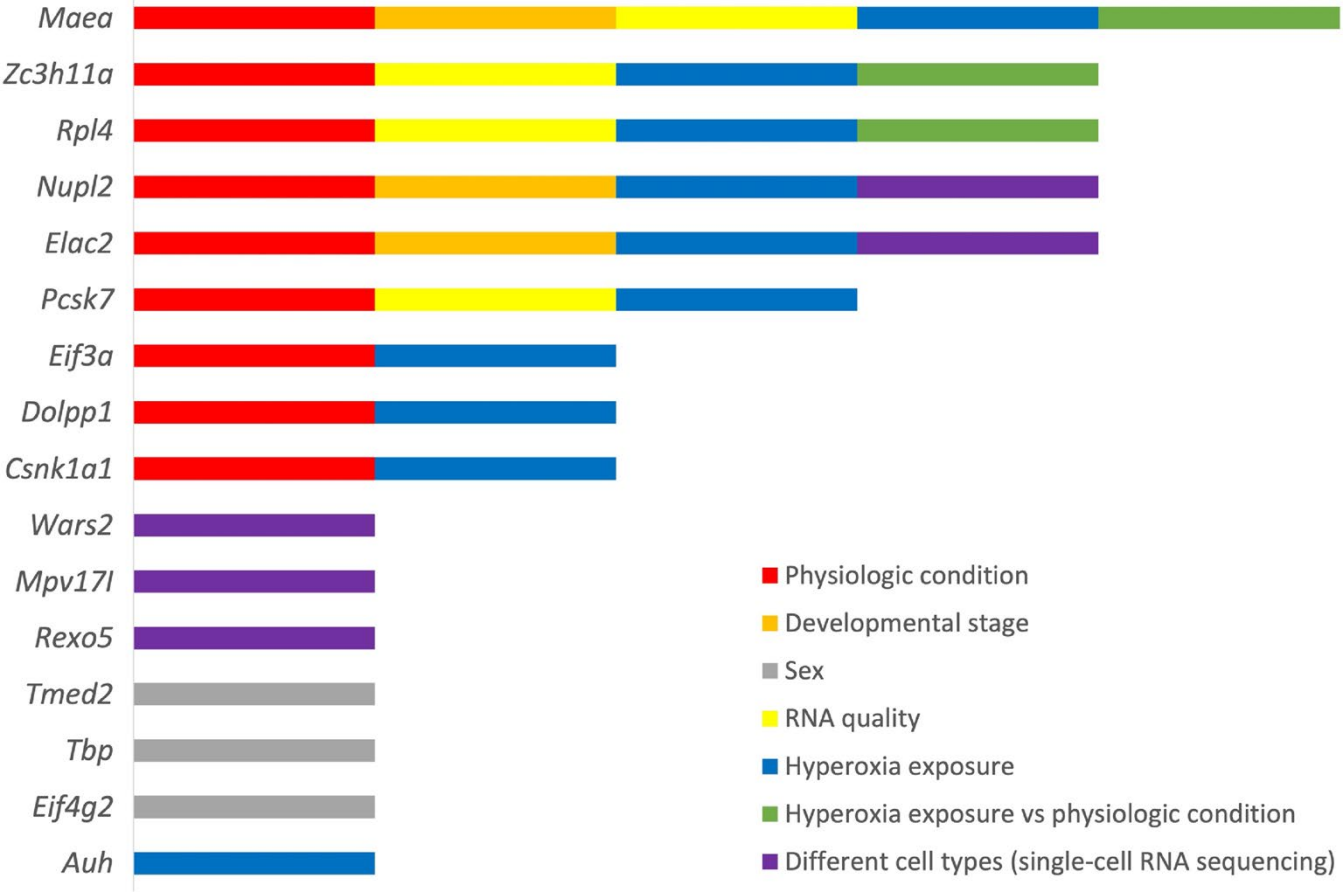
The reliable reference genes with regard to the analytical approaches. 16 out of 23 reference gene candidates that were identified in at least one analytical approach (colored bars) as a reliable reference gene are presented. *Maea* was most commonly identified as stably expressed gene among the different analytical approaches. For detailed information about experimental conditions of the analytical approaches see the text.

In summary, 16 out of 23 reference gene candidates can be suggested as ‘reliable’ based on their expression stability in at least one of the experimental settings investigated (Fig. 4). For the majority of genes such as *Elac2*, *Csnk1a1*, *Eif3a*, *Eif4g2*, *Tmed2* and *Mpv17l,* a role in the pathogenesis of lung diseases was indicated by previous studies^22–27^, although their functions in the neonatal lung remain unexplored. The data resource provided by the study not only gives insight into an unexplored field of significant relevance for studies in lung development but could serve as a guide for reference gene selection, specifically considering commonly relevant biological, technical and experimental conditions.

## Methods

After pre-selection of reference gene candidates by reviewing literature and publicly available databases including Genevestigator, we confirmed expression variability of the reference gene candidates using our own transcriptome data obtained from murine lungs during alveolarization [postnatal day (PND) 5–7] as well as microarray data available through Genevestigator. We validated the gene expression profiles of the final reference gene candidates by RT-qPCR in lung tissue of neonatal C57BL/6 considering different biological, technical and experimental conditions. These analyses were performed using GeNorm and Normfinder algorithms for the characterization of reference gene expression variability. Cell-specific expression patterns of the reference gene candidates were assessed using single-cell data obtained during postnatal lung development (PND0-PND14).

### In silico selection of reference gene candidates

Reference gene candidates were selected from four independent public and commercial databases (see **a–d**) taking into account studies that used both adult and neonatal mice as well as different experimental conditions in order to allow for broad coverage at the initial step. The search was limited by (i) underrepresentation of studies in neonatal mice in public and commercial databases, (ii) unavailability of sex-specific information and (iii) insufficient data on RNA quality for the majority of studies. The selection process combined reference gene candidates from the following four sources: (a) PubMed search using the terms ‘reference gene’, ‘murine lung’, ‘C57BL/6’, ‘neonatal’, ‘lung development’, ‘RT-qPCR’ and ‘housekeeping gene’ (single and combined search). Here, reference gene candidates were selected from the studies performed in lung tissue from C57BL/6 mice with the age from PND3 to 26 months. one study^5^ investigated neonatal mice between PND3 and PND21; (b) publications that specifically evaluated the expression variability of commonly-used murine reference genes including studies in C57BL/6, p50 and p105 transgenic mice aged 8–15 weeks^11–14^. Here, one meta-analysis of murine microarray data covered different murine strains, experimental conditions and age groups^11^; (c) the subtool ‘RefGenes’ of Genevestigator (https://genevestigator.com/gv) using the selection criteria ‘lung tissue’ and ‘C57BL/6’ resulting in 10 gene expression profiling studies from 2 to 22 week-old C57BL/6 mice (n = 122 wild-type; n = 17 transgenic mice; total n = 139 samples) with one study in 2 week old C57BL/6 mice^28^. These studies included different experimental conditions such as simvastatin treatment, cigarette smoke exposure and ovalbumin sensitization^15,28–36^. All genes identified with RefGenes at three different expression levels (log2-scaled signal intensity: > 13.00 high level, 10.00–13.00 medium level, < 10.00 low level; n = 20 each) were selected excluding doublet candidates; (d) a commercial reference gene catalogue (Roche Applied Science, n = 27 reference genes).

### Validation of reference gene expression variability using our own lung transcriptome data and Genevestigator

As the majority of gene candidates were derived from data obtained in adult mice, we next assessed their expression in neonatal murine lung tissues (PND 5–7) using own transcriptome data to then rank expression variability of all reference gene candidates according to the overall SD (*SD*_*overall*_). Overall SD, sensitively detecting variability, was calculated as the square root of the averaged variances of the mean expression values from our own neonatal mouse microarray data (*SD*_*array*_) and Genevestigator RefGene analysis (*SD*_*ref*_*)*, predominantly derived from adult mice:

$${SD}_{overall}= \sqrt{\frac{{{SD}_{ref}}^{2} + {{SD}_{array}}^{2}}{2}}.$$

### Investigation of pulmonary expression variability under consideration of biological, technical and experimental variables

RT-qPCR for selected reference gene candidates in lung tissue included C57BL/6 mice (n = 17) during lung development under physiologic conditions (FiO_2_ = 0.21) and good RNA quality [RNA Integrity Number (RIN) > 5] with alveolarization [PND2.5 (n = 6), PND5.5 (n = 7)] and post-alveolarization [PND14.5–PND28.5 (n = 4)] stages and subsequently addressed the impact of biological and technical conditions, i.e., sex and RNA quality^37^. Sex: Expression variability of the reference gene candidates was compared in male (PND2.5–28.5, RIN > 5; n = 21) and female (PND2.5–28.5, RIN > 5; n = 20) mice. RNA quality: Expression variability was analyzed under consideration of RNA quality comparing results in low (RIN < 5, PND2.5–28.5; n = 33) and high (RIN > 5, PND2.5–28.5; n = 17) RNA quality samples^37^ under physiologic conditions (FiO_2_ = 0.21). In a subsequent comparison, expression variability was evaluated in the experimental setting of hyperoxia (FiO_2_ = 0.8, PND2.5–28.5, RIN > 5; n = 36) and compared to results obtained under physiologic conditions (FiO_2_ = 0.21, PND2.5–28.5, RIN > 5; n = 17).

### Assessment of expression variability

Analyses of raw Cq data were performed using GeNorm and Normfinder integrated in GenEx 7.0 software (MultiD Analyses AB; https://multid.se/genex/). For each analysis group, GeNorm identified the two reference genes with similar gene expression by pairwise comparison, whereas Normfinder identified the reference gene based on intra- and intergroup gene expression variation as introduced by different conditions, e.g., biological or technical conditions^19,38^.

For identification of the candidates with lowest expression variabilities, GeNorm and Normfinder, were applied as follows: (i) When considering a biological variable or experimental condition, all reference gene candidates with M- or SV-values below 0.5 were regarded as reliable reference genes according to a previous study^16,17^ and ranked according to the lowest M- or SV-value. (ii) When comparing two or more biological, technical variables or experimental conditions, expression variability of the reference gene candidates was ranked according to M- or SV-values in each group and subsequently, the mean expression variability rank for each gene was calculated by averaging the ranks from each group for genes with M- or SV-values below 0.5 in all groups. (iii) For comparison of two groups by means of Normfinder, intra- and intergroup variation analysis with sufficient sample numbers was performed.

### In vivo experiments and mRNA analysis in neonatal murine lungs

#### Microarray experiments and data analysis

Microarray experiments were performed in lung tissue obtained from 5 to 7-day-old C57BL/6 mice (FiO_2_ = 0.21). Lungs were excised and immediately snap frozen in liquid nitrogen before total RNA was extracted from homogenized tissues using the miRNeasy Mini Kit (Qiagen). Extracted total RNA was subjected to cRNA synthesis, cRNA fragmentation and finally hybridization on Mouse CodeLink 10 K arrays using the CodeLink Expression Assay Kit (GE Healthcare) according to the manufacturer's instructions. The spot signals of obtained microarray images were quantified using the CodeLink System Software 5.0.0.31312 which generated local background corrected raw as well as median centered intra-slide normalized data. For further analysis only the intra-slide normalized data consisting of 10,181 probe sets and 6 samples were used.

The genes represented by the probe sets were annotated using the biocLite package (BioConductor) with the library “mwgcod.db” for CodeLink Mouse Whole Genome arrays. The expression data were processed using an automated quality control workflow which includes omission of control genes, removal of genes with poor quality flags and removal of probe sets with high proportion (≥ 50%) of missing values. A total of 3651 probe sets remained after quality control. Replicates were averaged by calculating the mean if applicable and remaining missing values were imputed by sequential KNN imputation using SeqKnn Vers. 1.0. Imputed dataset was quantile normalized using the normalizeQuantiles from the limma package. Finally, logarithm for the base 2 was calculated. Microarray data conform to the MIAME standard^39^ and have been deposited in NCBI´s Gene Expression Omnibus (https://www.ncbi.nlm.nih.gov/geo/) and are accessible through GEO Series accession number GSE189505.

#### RT-qPCR experiments and data analysis

Wild-type male and female mice (C57BL/6J) were randomly assigned to the experimental groups to spontaneously breath room air (FiO2 = 0.21) or undergo hyperoxia exposure while spontaneously breathing (FiO2 = 0.8). Total RNA was extracted from homogenized whole lungs using the PeqGOLD total RNA kit (PeqLab). Samples were stored at − 80 °C until further analysis. *Real time qPCR:* RNA quality was assessed using the RNA 6000 Nano Kit (Agilent Technologies) and RT-qPCR was then performed according to the manufacturer’s instructions. Briefly summarized, equivalents of 1 µg total RNA were transcribed into cDNA (Transcriptor First Strand cDNA synthesis kit, Roche) and subsequently diluted 1:10 using PCR-grade water. All 24 reference gene candidates were assessed in the lung samples by RT-qPCR using FastStart essential DNA probes master (Roche) and RealTime Ready Assays (Roche) providing gene specific primers. For data analysis, inter-run differences were adjusted for by calibration using a fixed cDNA mix generated from 31 cDNA samples with the best RNA quality from each age group (RIN = 5.4–8.1). The cDNA mix was applied to every plate with the expression level of the gene *Tbp* (Entrez ID 21374) serving as an inter-run calibrator. Differences between plates were compensated through the inter-run calibrated Cq value using the formula demonstrated in the study of Ståhlberg et al.^40^.

Sex was confirmed using Y chromosome identification by qPCR (60 samples of 86 samples in total). Age groups were identified as neonatal, i.e., during alveolarization (PND2.5 and PND5.5) and adolescent/young adult, i.e., post-alveolarization (PND14.5–PND28.5).

### Single-cell RNA-sequencing analysis

Data from single-cell RNA sequencing in lung tissue samples were analyzed for marker gene expression in different lung cell types as previously reported^18^. This dataset was generated from a mouse lung cellular suspension that was depleted of Ter119 + blood cells and CD45 + immune cells and sequenced using the 10× Genomics Chromium platform. Data from P0, P3, P5, P7 and P14 mice were analyzed. Variability of marker genes across individual cells in the lung was calculated by determining the standard deviation of each gene across all cells in the presented dataset. Genes with the lowest standard deviation are considered the least variable.

### Approval for animal experiments

The study was conducted in accordance with the German animal welfare law (TierSchG and TierSchVersV) and the European legislation for the protection of animals used for scientific purposes (2010/63/EU). All animal experiments were approved by the Ethic-Commission of the Medical Faculty of the Justus-Liebig-University Giessen (Approval No. TVA B2/277) and the Institution for Animal Care, Ludwig-Maximilians-University Munich (Approval No. TVA 117-10). The study complies with the ARRIVE guidelines.

## Supplementary Information

Supplementary Information 1.

Supplementary Information 2.

## Data Availability

The microarray data generated during this study have been deposited in NCBI´s Gene Expression Omnibus (https://www.ncbi.nlm.nih.gov/geo/) and are accessible through GEO Series accession number GSE189505. The raw data obtained from RT-qPCR validation were provided as supplemental data.
