# Supplementary material for: Reference genes for the developing mouse lung under consideration of biological, technical and experimental confounders
Source: Sci Rep. 2022 Oct 21;12:17679. doi: 10.1038/s41598-022-19071-1 (PMC9587035; doi:10.1038/s41598-022-19071-1)
Supplement: Supplementary file 1 — Supplementary Information 1. [file 41598_2022_19071_MOESM1_ESM.docx]

**Supplementary data**

**Table S1 The 122 potential reference gene candidates identified in the pre-selection process.** The 122 potential reference gene candidates were first screened from four independent data sources, namely studies employing commonly used reference genes (step a), studies evaluating suitablity of commonly used reference genes and suggesting novel reliable ones (step b), Genevestigator® analyzing publicly available microarray data (step c) and Roche commercial panel of reference genes in frequent use. Afterwards, 76 genes were excluded from the further process with the criteria listed below and it remained a total of 46 candidate genes for further evaluation process. The candidate genes with the exclusion criteria "undetectable in our own microarray" (35 genes) were not included on CodeLink UniSet Mouse I Bioarray 10K platform used for establishing our own transcriptome data from neonatal mice. However, for 24 genes of them, other similar genes in the same gene family were tested by the CodeLink platform with absent expression in the investigated lung samples. These 35 genes without expression data in the neonatal murine lung tissues were thus excluded from the selection of final reference gene candidates.

| Index | Gene symbol | Entrez ID | Step | Reference for selection | Exclusion criteria |
| --- | --- | --- | --- | --- | --- |
|  |  |  |  |  |  |
| 1 | *Actb* | 11461 | b | Stoeger et al. 2010 | overlap between different data sources |
| 2 | *B2m* | 12010 | b | Shimada et al. 2009 | overlap between different data sources |
| 3 | *Gapdh* | 14433 | b | Shimada et al. 2009 | overlap between different data sources |
| 4 | *Gusb* | 110006 | b | Stoeger et al. 2010 | overlap between different data sources |
| 5 | *Oaz1* | 18245 | b | De Jonge et al. 2007 | overlap between different data sources |
| 6 | *Actb* | 11461 | d | Roche reference gene panel | overlap between different data sources |
| 7 | *B2m* | 12010 | d | Roche reference gene panel | overlap between different data sources |
| 8 | *Gapdh* | 14433 | d | Roche reference gene panel | overlap between different data sources |
| 9 | *Gusb* | 110006 | d | Roche reference gene panel | overlap between different data sources |
| 10 | *Hmbs* | 15288 | d | Roche reference gene panel | overlap between different data sources |
| 11 | *Pgk1* | 18655 | d | Roche reference gene panel | overlap between different data sources |
| 12 | *Ipo8* | 320727 | d | Roche reference gene panel | overlap between different data sources |
| 13 | *Ppia* | 268373 | d | Roche reference gene panel | overlap between different data sources |
| 14 | *Rpl13a* | 22121 | d | Roche reference gene panel | overlap between different data sources |
| 15 | *Oaz1* | 18245 | a | Rothman et al. 2011, De Jonge et al. 2007 | presence of pseudogene |
| 16 | *Eef2* | 13629 | b | Kouadjo et al. 2007 | presence of pseudogene |
| 17 | *Rpl22* | 19934 | b | De Jonge et al. 2007 | gene coding for ribosomal protein,^13^ presence of pseudogene |
| 18 | *Rpl27* | 19942 | b | De Jonge et al. 2007 | gene coding for ribosomal protein, presence of pseudogene |
| 19 | *Rpl37* | 67281 | b | Shimada et al. 2009, Kouadjo et al. 2007 | gene coding for ribosomal protein, presence of pseudogene |
| 20 | *Rpl38* | 67671 | b | Kouadjo et al. 2007 | gene coding for ribosomal protein, presence of pseudogene |
| 21 | *Rpl9* | 20005 | b | De Jonge et al. 2007 | gene coding for ribosomal protein, presence of pseudogene |
| 22 | *Rplp2* | 67186 | b | Shimada et al. 2009 | gene coding for ribosomal protein, presence of pseudogene |
| 23 | *Rps12* | 20042 | b | De Jonge et al. 2007 | gene coding for ribosomal protein, presence of pseudogene |
| 24 | *Rps13* | 68052 | b | De Jonge et al. 2007 | gene coding for ribosomal protein, presence of pseudogene |
| 25 | *Rps16* | 20055 | b | De Jonge et al. 2007 | gene coding for ribosomal protein, presence of pseudogene |
| 26 | *Rps20* | 67427 | b | De Jonge et al. 2007 | gene coding for ribosomal protein, presence of pseudogene |
| 27 | *Hbb-b1* | 15129 | d | Roche reference gene panel | expected low expression stability in hyperoxia as subunit of oxygen carrier |
| 28 | *B2m* | 12010 | a | Tzouvelekis et al. 2009 | not expressed in our own microarray data |
| 29 | *Hmbs* | 15288 | a | Kitowska et al. 2008 | not expressed in our own microarray data |
| 30 | *Pgk1* | 18655 | a | Yamaji et al. 2010 | not expressed in our own microarray data |
| 31 | *Chmp2a* | 295670 | b | Kouadjo et al. 2007 | not expressed in our own microarray data |
| 32 | *Heatr3* | 234549 | b | Kouadjo et al. 2007 | undetectable in our own microarray data |
| 33 | *Ipo8* | 320727 | b | Shimada et al. 2009 | undetectable in our own microarray data |
| 34 | *Leng8* | 232798 | b | Kouadjo et al. 2007 | undetectable in our own microarray data |
| 35 | *Ppia* | 268373 | b | Stoeger et al. 2010 | not expressed in our own microarray data |
| 36 | *Rpl24* | 68193 | b | De Jonge et al. 2007 | undetectable in our own microarray data |
| 37 | *Rpl6* | 19988 | b | De Jonge et al. 2007 | not expressed in our own microarray data |
| 38 | *Rps29* | 20090 | b | De Jonge et al. 2007 | not expressed in our own microarray data |
| 39 | *Srp14* | 20813 | b | De Jonge et al. 2007 | undetectable in our own microarray data |
| 40 | *Rpl13a* | 22121 | b | Stoeger et al. 2010, De Jonge et al. 2007 | not expressed in our own microarray data |
| 41 | *Heatr7a* | 223658 | c | Genevestigator® | undetectable in our own microarray data |
| 42 | *Maskbp3* | 71783 | c | Genevestigator® | undetectable in our own microarray data |
| 43 | *Zfp182* | 319535 | c | Genevestigator® | undetectable in our own microarray data |
| 44 | *1446253_at* | n/a | c | Genevestigator® | undetectable in our own microarray data |
| 45 | *Cep57l1* | 103268 | c | Genevestigator® | undetectable in our own microarray data |
| 46 | *Il15ra* | 16169 | c | Genevestigator® | undetectable in our own microarray data |
| 47 | *Ints5* | 109077 | c | Genevestigator® | undetectable in our own microarray data |
| 48 | *LOC106740* | 106740 | c | Genevestigator® | undetectable in our own microarray data |
| 49 | *Metap1* | 75624 | c | Genevestigator® | undetectable in our own microarray data |
| 50 | *Nek4* | 23955 | c | Genevestigator® | not expressed in our own microarray data |
| 51 | *Nup214* | 227720 | c | Genevestigator® | undetectable in our own microarray data |
| 52 | *Plekhm3* | 241075 | c | Genevestigator® | undetectable in our own microarray data |
| 53 | *Sec62* | 69276 | c | Genevestigator® | undetectable in our own microarray data |
| 54 | *Ywhaq* | 22630 | c | Genevestigator® | undetectable in our own microarray data |
| 55 | *Zfp592* | 233410 | c | Genevestigator® | undetectable in our own microarray data |
| 56 | *Zxda* | 668166 | c | Genevestigator® | not expressed in our own microarray data |
| 57 | *Gabpb2* | 213054 | c | Genevestigator® | undetectable in our own microarray data |
| 58 | *Mcph1* | 244329 | c | Genevestigator® | undetectable in our own microarray data |
| 59 | *Nudt6* | 229228 | c | Genevestigator® | undetectable in our own microarray data |
| 60 | *Trim37* | 68729 | c | Genevestigator® | undetectable in our own microarray data |
| 61 | *Zfp251* | 71591 | c | Genevestigator® | undetectable in our own microarray data |
| 62 | *1110035M17Rik* | 432396 | c | Genevestigator® | undetectable in our own microarray data |
| 63 | *1435789_x_at* | n/a | c | Genevestigator® | undetectable in our own microarray data |
| 64 | *Caprin2* | 232560 | c | Genevestigator® | undetectable in our own microarray data |
| 65 | *Crnkl1* | 66877 | c | Genevestigator® | not expressed in our own microarray data |
| 66 | *Gtf2h3* | 209357 | c | Genevestigator® | undetectable in our own microarray data |
| 67 | *H3f3a* | 15078 | c | Genevestigator® | undetectable in our own microarray data |
| 68 | *Hnrnph1* | 59013 | c | Genevestigator® | undetectable in our own microarray data |
| 69 | *Rplp0* | 11837 | d | Roche reference gene panel | undetectable in our own microarray data |
| 70 | *Trpt1* | 107328 | d | Roche reference gene panel | undetectable in our own microarray data |
| 71 | *Alas2* | 11656 | d | Roche reference gene panel | undetectable in our own microarray data |
| 72 | *Cyp2d13* | 68444 | d | Roche reference gene panel | not expressed in our own microarray data |
| 73 | *Fxyd6* | 59095 | d | Roche reference gene panel | not expressed in our own microarray data |
| 74 | *Fxyd7* | 57780 | d | Roche reference gene panel | undetectable in our own microarray data |
| 75 | *H2-Ea-ps* | 100504404 | d | Roche reference gene panel | not expressed in our own microarray data |
| 76 | *Oas1b* | 23961 | d | Roche reference gene panel | undetectable in our own microarray data |
| 77 | *Actb* | 11461 | a | Yamaji et al. 2010 | - |
| 78 | *Gapdh* | 14433 | a | Sakazaki et al. 2011, Yamaji et al. 2010 | - |
| 79 | *Hsp90ab1* | 15516 | d | Roche reference gene panel | - |
| 80 | *Pla2g2a* | 18780 | d | Roche reference gene panel | - |
| 81 | *Gusb* | 110006 | a | McDonald et al. 2008 | - |
| 82 | *Rhot1* | 59040 | c | Genevestigator® | - |
| 83 | *Ing3* | 71777 | c | Genevestigator® | - |
| 84 | *G6pdx* | 14381 | d | Roche reference gene panel | - |
| 85 | *Sdha* | 66945 | d | Roche reference gene panel | - |
| 86 | *Hdgfrp2* | 15193 | c | Genevestigator® | - |
| 87 | *Tomm20* | 67952 | c | Genevestigator® | - |
| 88 | *Mx2* | 17858 | d | Roche reference gene panel | - |
| 89 | *Atp6v0c* | 11984 | c | Genevestigator® | - |
| 90 | *Ywhaz* | 22631 | d | Roche reference gene panel | - |
| 91 | *Eif4h* | 22384 | c | Genevestigator® | - |
| 92 | *Slc35a5* | 74102 | c | Genevestigator® | - |
| 93 | *Canx* | 12330 | c | Genevestigator® | - |
| 94 | *Myadm* | 50918 | c | Genevestigator® | - |
| 95 | *Rrp1b* | 72462 | c | Genevestigator® | - |
| 96 | *Hprt* | 15452 | d | Roche reference gene panel | - |
| 97 | *Rab23* | 19335 | c | Genevestigator® | - |
| 98 | *F2r* | 14062 | c | Genevestigator® | - |
| 99 | *Rpl4* | 67891 | b | De Jonge et al. 2007 | - |
| 100 | *Psmd4* | 19185 | b | Kouadjo et al. 2007 | - |
| 101 | *Tfrc* | 22042 | d | Roche reference gene panel | - |
| 102 | *Puf60* | 67959 | b | Kouadjo et al. 2007 | - |
| 103 | *Csnk1a1* | 93687 | c | Genevestigator® | - |
| 104 | *Tnks2* | 74493 | c | Genevestigator® | - |
| 105 | *Eif4g2* | 13690 | c | Genevestigator® | - |
| 106 | *Eif3a* | 13669 | b | Kouadjo et al. 2007 | - |
| 107 | *Tbp* | 21374 | d | Roche reference gene panel | - |
| 108 | *Grn* | 14824 | c | Genevestigator® | - |
| 109 | *Tmed2* | 56334 | c | Genevestigator® | - |
| 110 | *Nupl2* | 231042 | c | Genevestigator® | - |
| 111 | *Rexo5* | 434234 | c | Genevestigator® | - |
| 112 | *Zc3h11a* | 70579 | c | Genevestigator® | - |
| 113 | *Efhd2* | 27984 | c | Genevestigator® | - |
| 114 | *Auh* | 11992 | c | Genevestigator® | - |
| 115 | *Wars2* | 70560 | c | Genevestigator® | - |
| 116 | *Fkbp1a* | 14225 | c | Genevestigator® | - |
| 117 | *Maea* | 59003 | c | Genevestigator® | - |
| 118 | *Kdm3b* | 277250 | c | Genevestigator® | - |
| 119 | *Elac2* | 68626 | c | Genevestigator® | - |
| 120 | *Pcsk7* | 18554 | c | Genevestigator® | - |
| 121 | *Mpv17l* | 93734 | c | Genevestigator® | - |
| 122 | *Dolpp1* | 57170 | c | Genevestigator® | - |
